# Plant characterization of insect-protected soybean

**DOI:** 10.1007/s11248-024-00391-z

**Published:** 2024-06-20

**Authors:** Duška Stojšin, Hallison Vertuan, Chen Meng, Roger Effertz, Marcia Jose, Debbie Mahadeo, Augusto Crivellari, Christy Hu, Geraldo Berger

**Affiliations:** 1 Bayer Crop Science, St. Louis, MO USA; 2 Bayer Crop Science, São Paulo, Brazil; 3 Bayer Crop Science, Beijing, China

**Keywords:** Genetically modified crops, Environmental risk assessment, Plant characterization, Data transportability

## Abstract

Insect-protected soybean (SIP) that produces the Cry1A.105 and Cry2Ab2 insecticidal crystal proteins has been developed to provide protection from feeding damage caused by targeted lepidopteran insect pests. Typically, as part of environmental risk assessment (ERA), plant characterization is conducted, and the data submitted to regulatory agencies prior to commercialization of genetically modified (GM) crops. The objectives of this research were to: (a) compare soybean with and without the SIP trait in plant characterization field trials designed to fulfill requirements for submissions to global regulatory agencies and address China-specific considerations and (b) compare risk assessment conclusions across regions and the methodologies used in the field trials. The soybean with and without the SIP trait in temperate, tropical, and subtropical germplasm were planted in replicated multi-location trials in the USA (in 2012 and 2018) and Brazil (in 2013/2014 and 2017/2018). Agronomic, phenotypic, plant competitiveness, and survival characteristics were assessed for soybean entries with and without the SIP trait. Regardless of genetic background, growing region, season, or testing methodology, the risk assessment conclusions were the same: the evaluated insect-protected soybean did not differ from conventional soybean in evaluated agronomic, phenotypic, competitiveness, and survival characteristics indicating no change in plant pest/weed potential. These results reinforce the concept of data transportability across global regions, different seasons, germplasm, and methodologies that should be considered when assessing environmental risks of GM crops.

## Introduction

Genetically modified (GM) crops have been commercialized for over 25 years providing herbicide tolerance, insect resistance, and/or nutritionally enhanced crops without compromising food and feed security (ISAAA 2018). Prior to their commercialization, an extensive environmental risk assessment (ERA) is conducted, and results are submitted to regulatory agencies for food and feed import and/or cultivation approval of GM crops. As part of the ERA, field trials are conducted with the objective of making comparisons between GM and non-GM counterparts. If entries with and without the GM trait show comparable performance, then there is no plausible hypothesis for the GM crop becoming more weedy or invasive than its conventional counterpart control (Anderson et al. 2021). Numerous studies have demonstrated that evaluated GM products represent no greater risk to the environment than their conventional counterparts (Bell et al. 2018; Berman et al. 2011; de Cerqueira et al. 2017; Gampala et al. 2017; Herman et al. 2017; Marques et al. 2018; Raybould et al. 2012; Ridley et al. 2011; Venkatesh et al. 2014).

Under the concept of data transportability, field trials conducted as part of comparative assessments are utilized to support the GM approval process across different regions. However, if data transportability is not fully accepted or there are differences among regions in regulatory requirements, then field trials must be repeated in multiple regions.

Agronomic and phenotypic characteristics assessed as part of ERA for GM products are those typically considered by breeders and agronomists (EFSA 2015). Historically, when observing these common breeding parameters, breeders and agronomists have not developed any crop variety that showed more weediness or increased plant pest potential. Thus, observing similar agronomic and phenotypic characteristics in GM crops and showing that their performance is comparable to non-GM control is a good indicator that unintended weediness or plant pest potential has not changed with the introduction of the GM trait. This approach of testing common agronomic and phenotypic characteristics as part of ERA has been accepted by most global regulatory agencies. Furthermore, with over two decades of experience with GM crop production, many regulatory agencies have accepted results generated by observation of common agronomic and phenotypic characteristics in a different country/region, following the data transportability approach.

However, some regional regulatory agencies might require additional data or different methodology as part of their risk assessment. For example, although China’s regulatory authority has begun exempting in-country field trials for import purposes, if field trials are requested, the requirements include assessments of GM soybean under both cultivated and uncultivated conditions. These studies evaluate plant competitiveness and survival of GM soybean compared to the conventional control and require comprehensive evaluation of weed and soybean characteristics both during and after the growing season.

The current research is based on several field trials that were conducted to assess characteristics associated with agronomic, phenotypic, competitiveness, and survival of soybean insect-protected (SIP) trait in different genetic backgrounds, across regions, seasons, and using different methodologies. Comparable conclusions would reinforce the concept of data transportability across regions, seasons, germplasm, and methodologies and should be considered when assessing environmental risks of transgenic crops. Thus, the objectives of this research were to: (a) compare soybean with and without the SIP trait in plant characterization field trials set to fulfill requirements for submissions to global regulatory agencies and address China-specific considerations and (b) compare risk assessment conclusions across regions and the methodologies used in the field trials.

## Materials and methods

The SIP trait evaluated in this research was biotechnology-derived MON 87751 soybean developed as a single-vector transgenic event that produces the Cry1A.105 and Cry2Ab2 insecticidal crystal (Cry) proteins (δ-endotoxin) derived from *Bacillus thuringiensis*. These proteins provide protection against feeding damage caused by targeted lepidopteran insect pests. The soybean entries evaluated in this research included varieties with and without the SIP trait across three different maturity groups (MG 3.5, 5.9, and 8.3).

### Cultivated field trials

Field trials under cultivated conditions were conducted as part of plant characterization assessment for global submissions to regulatory agencies. The trials were set in a randomized complete block design (RCBD) with four replications in 2012 in the USA (temperate climate) and during 2013/2014 growing seasons in Brazil (subtropical and tropical climate). The entries with and without the SIP trait in MG 3.5 background were grown across 17 locations representing 12 USA states including Arkansas (1), Georgia (1), Iowa (3), Illinois (4), Kansas (1), Louisiana (1), Missouri (1), North Carolina (1), South Carolina (1), Nebraska (1), Ohio (1), and Pennsylvania (1) (number of locations in parentheses). In Brazil, tropical (MG 8.3) and subtropical (MG 5.9) varieties were evaluated across a total of six locations. The entries with and without the SIP trait in MG 8.3 background were grown across three states including Mato Grosso (1), Minas Gerais (1), and Bahia (1). The entries with and without the SIP trait in MG 5.9 background were grown across three states including São Paulo (1), Paraná (1), and Rio Grande do Sul (1). The 6-row plots were 6 m long and 4.6 m wide in the USA. The 8-row plots were 6 m long and 4 m wide in Brazil. The characteristics typically evaluated for global submissions to regulatory (EFSA 2015) agencies are summarized in Table 1.

**Table 1:** Soybean characteristics evaluated in the USA and Brazil field trials conducted in accordance with global regulatory submission requirements

Characteristic (unit)	Timing	Description
Early stand count (plants/m)	V2–V4	Number of plants per linear meter
Days to flowering	R1	Number of days from planting to 50% flowering per plot
Plant height (cm)	R8	Distance from the soil surface to the uppermost node on the main stem measured on 5 representative plants per plot
Plant lodging (1–9)	R8	Lodging was rated using the 1–9 scale, where 1 = completely upright plants (no lodging) and 9 = completely lodged plants
Pod shattering (1–9)	R8	Shattering was rated using the 1–9 scale, where 1 = no shattering and 9 = completely shattered pods
Final stand count (plants/m)	R8	Number of plants per linear meter
Grain moisture (%)	R8	Moisture of harvested grain
100 Seed weight (g)	R8	Weight of 100 seeds standardized to 13% moisture
Yield (t/ha)	R8	Grain yield standardized to 13% moisture

Additional field trials under cultivated conditions were conducted as part of soybean plant characterization assessment in accordance with China Ministry of Agriculture specifications (MARA 2013). Entries with and without the SIP trait were planted at two locations: one in Illinois, USA during 2018 and the other in Rio Grande do Sul, Brazil during 2017/2018 season. Trials were established in RCBD with 4 replications. The plots were 5 m long and 4 m wide. Planting was done in 6-row plots in the USA and 8-row plots in Brazil. The characteristics were evaluated according to China-specific considerations and summarized in Table 2.

**Table 2:** Soybean and weed characteristics evaluated in the USA and Brazil field trials conducted in accordance with China-specific regulatory submission considerations

Characteristic (unit)	Timing	Description
*Cultivated Field Trials*		
Soybean early stand count (plants/m)	V3	Number of soybean plants per linear meter
Soybean plant height (cm)	V3, V5, R1, R3, R6	Distance from the soil surface to the uppermost node on the main stem measured on 10 representative plants per plot
Soybean canopy coverage (%)	V3, V5, R1, R3, R6	Proportion of soybean canopy coverage visually estimated per plot
Soybean leaf number	V3, V5, R1, R3, R6	Number of trifoliate leaves was determined in 10 plants per plot
Soybean final stand count (plants/m)	R8	Number of soybean plants per linear meter
Soybean seed shattering (%)	R8	Cumulative shattering percentage across 5 observation times on 10 plants per plot
Soybean seed number per plant	R8	Number of seeds per plant measured on 10 plants per plot
Soybean shattered seed count (seeds/plot)	After harvest	Total number of shattered seeds per plot
Soybean volunteer plants (plants/plot)	After harvest	Number of soybean volunteer plants that emerged the following season
*Uncultivated Field Trials*		
Soybean stand count (plants/plot)	30, 60, 90, 120 DAP^1^	Number of soybean plants per plot
Soybean plant height (cm)	30, 60, 90, 120 DAP	Distance from the soil surface to the uppermost node on the main stem measured on 10 representative plants per plot
Soybean canopy coverage (%)	30, 60, 90, 120 DAP	Proportion of soybean canopy coverage visually estimated per plot
Soybean seed number per plant	R8	Number of seeds per plant measured on 10 plants per plot
Soybean volunteer plants (plants/plot)	After harvest	Number of soybean volunteer plants that emerged the following season
Weed stand count (plants/plot)	Pre-soybean planting, 30, 60, 90, 120 DAP	Number of plants of the dominant weed species per plot
Weed plant height (cm)	Pre-soybean planting, 30, 60, 90, 120 DAP	Distance from the soil surface to the plant tip measured on 10 representative plants of the dominant weed species per plot
Weed canopy coverage (%)	Pre-soybean planting, 30, 60, 90, 120 DAP	Proportion of weed canopy coverage visually estimated per plot

^1^DAP–Days after planting

In these trials conducted under cultivated conditions, crop management practices typical for the region were implemented uniformly across all plots at each site. Considering that the trials were set to evaluate agronomic and phenotypic performance, special attention was paid to monitoring and protecting (if needed) against insects controlled by MON 87751 soybean. This ensured that the control entry without SIP trait was protected against feeding damage caused by targeted lepidopteran insects.

### Uncultivated field trials

Trials under uncultivated conditions consistent with China Ministry of Agriculture specifications (MARA 2013) were planted at two locations; one in Illinois, USA during 2018 and the other one in Rio Grande do Sul, Brazil during the 2017/2018 season. In the USA, the field trial was established in an uncultivated, weedy area that had been left fallow, with no chemical treatments for 18 months and no agronomic maintenance for 12 months except for periodic mowing. In Brazil, the trial was established in a field that was covered with weedy vegetation and had been without chemical application for over 10 months. At each location, the trials were planted at four different times utilizing two planting methods (surface scattered and row seeded) with 300 soybean seeds per plot. The trials were established in RCBD with four replications. Individual plots were 4 m long and 4 m wide with at least 0.5 m alleys between plots. In the USA, the trial was planted on April 24, May 18, June 13, and July 11, 2018. In Brazil, the trial was planted on September 13, October 15, November 15, and December 15, 2017. All plots were left undisturbed and unmanaged throughout the course of the research except during data collection. Plant characteristics of soybean and weeds were observed across all plots several times during the season, according to China-specific considerations. The evaluated characteristics are summarized in Table 2.

### Statistical analyses

*Cultivated Field Trials:* Agronomic and phenotypic characteristics of entries with and without the SIP trait were statistically compared in the USA (2012) and Brazil (2013/2014) field trials established to collect plant characteristics in accordance with requirements for global regulatory submissions (EFSA 2015). Goodness-of-fit of the linear mixed models used in the analysis was evaluated by visual examination of residual plots and histograms. Based on the evaluation, data transformation and/or unequal residual variance were applied as appropriate to satisfy the normality and homogeneity assumptions. Combined-site analysis was conducted according to the following linear mixed model:

1 $${\text{Y}}_{{{\text{ijk}}}} = \mu + {\text{E}}_{{\text{i}}} + {\text{S}}_{{\text{k}}} + \left( {{\text{ES}}} \right)_{{{\text{ik}}}} + {\text{R}}\left( {\text{S}} \right)_{{{\text{j}}\left( {\text{k}} \right) }} + \varepsilon_{{{\text{ijk}}}}$$

where Y_ijk_ is the observed value of the characteristic, µ is the overall mean, E_i_ is the fixed SIP event effect, S_k_ is the random site effect, R(S)_j(k)_ is the random within-site replicate effect, (ES)_ik_ is the random interaction of site with SIP event, and ε_ijk_ represents the residual effect. SAS PROC MIXED (SAS 2012) was used to fit model (1) separately for each characteristic within each field study. Pair-wise comparisons between SIP and conventional control were defined within model (1) and tested using t-tests.

Furthermore, analysis was also conducted for the data in the USA (2018) and Brazil (2017/2018) field trials established in accordance with China-specific regulatory considerations, according to the following linear mixed model:

2 $${\text{Y}}_{{{\text{ij}}}} = \mu + {\text{E}}_{{\text{i}}} + {\text{R}}_{{\text{j}}} + \varepsilon_{{{\text{ij}}}}$$

where Y_ij_ is the observed value of the characteristic, µ is the overall mean, E_i_ is the fixed SIP event effect, R_j_ is the random replicate effect, and ε_ij_ represents the residual effect. SAS PROC MIXED was used to fit model (2) for each site and characteristic within each field study. Pair-wise comparisons between SIP and control materials were defined within model (2) and tested using t-tests.

*Uncultivated Field Trials:* Analyses of evaluated plant characteristics were conducted for the data collected from the USA (2018) and Brazil (2017/2018) uncultivated field trials established in accordance with China-specific regulatory considerations, according to the following linear mixed model:

3 $${\text{Y}}_{{{\text{ijklm}}}} = \mu + {\text{M}}_{{\text{i}}} + {\text{T}}_{{\text{j}}} + {\text{C}}_{{\text{k}}} + {\text{O}}_{{\text{l}}} + {\text{E}}_{{\text{m}}} + {\text{MT}}_{{{\text{ij}}}} + {\text{MC}}_{{{\text{ik}}}} + {\text{ME}}_{{{\text{im}}}} + {\text{TC}}_{{{\text{jk}}}} + {\text{TE}}_{{{\text{jm}}}} + {\text{CE}}_{{{\text{km}}}} + \varepsilon_{{{\text{ijklm}}}}$$

where Y_ijklm_ is the observed response for the combination of ith planting method, jth planting time, kth country, lth observation time, and mth event; μ represents the overall mean response; M_i_ represents the fixed effect of the ith planting method; T_j_ represents the fixed effect of the jth planting time; C_k_ represents the fixed effect of the kth country; O_l_ represents the random effect of the lth observation time; E_m_ represents the fixed effect of the mth event; MT_ij_, MC_ik_, ME_im_, TC_jk_, TE_jm_, and CE_km_ represent the interactions between the fixed effects; and ε_ijklm_ represents the residual error. SAS PROC MIXED was used to fit model (3), with all the default options of the procedure unless otherwise noted, for each soybean and weed characteristic. An appropriately reduced linear mixed model (4), excluding the random effect of observation time, was used to conduct the analysis for the soybean seed number and volunteer plants characteristics.

4 $${\text{Y}}_{{{\text{ijkm}}}} = \mu + {\text{M}}_{{\text{i}}} + {\text{T}}_{{\text{j}}} + {\text{C}}_{{\text{k}}} + {\text{E}}_{{\text{m}}} + {\text{MT}}_{{{\text{ij}}}} + {\text{MC}}_{{{\text{ik}}}} + {\text{ME}}_{{{\text{im}}}} + {\text{TC}}_{{{\text{jk}}}} + {\text{TE}}_{{{\text{jm}}}} + {\text{CE}}_{{{\text{km}}}} + \varepsilon_{{{\text{ijkm}}}}$$

where Y_ijkm_ is the observed response for the combination of ith planting method, jth planting time, kth country, and mth event; μ represents the overall mean response; M_i_ represents the fixed effect of the ith planting method; T_j_ represents the fixed effect of the jth planting time; C_k_ represents the fixed effect of the kth country; E_m_ represents the fixed effect of the mth event; MT_ij_, MC_ik_, ME_im_, TC_jk_, TE_jm_, and CE_km_ represent the interactions between the fixed effects; and ε_ijkm_ represents the residual error.

In order to satisfy the model assumptions, a square-root transformation was used for the count data and an arcsine transformation was used on the square-root of the canopy coverage proportions. To accommodate the heterogeneity of the residual variation between countries, the residual variances of the USA and Brazil were estimated with two different variance components using the REPEATED statement in the MIXED procedure.

For each soybean and weed characteristic observed multiple times during the season (stand count, plant height, and canopy coverage), correlation analyses were done between the SIP and control plots. The coefficient of correlation (*r*) was calculated and provided a measure of the linear relationship between the SIP and control plots.

To assess the different sources of variation, a variance component analysis was conducted by treating all effects in models (3) and (4) as random. Variance was estimated for each effect in models (3) and (4), which represents the amount of variability in the responses associated with those effects. The percentages of total variability attributable to the main effects of planting method, planting time, country, and event, as well as the other effects (observation time, interactions, residual) were estimated for the soybean and weed characteristics.

## Results

### Cultivated field trials

Plant characterization under cultivated field trials showed no significant differences between soybean entries with and without the SIP trait for any of the evaluated characteristics, regardless of testing region or whether they were tested in temperate, subtropical, or tropical genetic backgrounds. This was observed for characteristics typically collected as part of plant ERA for submissions to global regulatory agencies (Table 3), as well as those collected in trials conducted in accordance with China-specific regulatory considerations (Table 4).

**Table 3:** Plant characterization of insect-protected soybean (SIP) compared to conventional control conducted in the USA and Brazil field trials in accordance with global regulatory submission requirements

Testing regions	USA		Brazil			
	2012 (17 Sites)^a^		2013/2014 (3 Sites)^b^		2013/2014 (3 Sites)^c^	
Phenotypic characteristic	SIP	Control	SIP	Control	SIP	Control
Early stand count (plants/m)	23.5	24.3	14.2	14.4	12.5	11.7
Plant height (cm)	85.4	83.9	64.5	62.4	102.8	106.7
Days to flowering	44.0	43.6	47.0	47.0	39.7	40.2
Plant lodging (1–9)	2.4	2.2	1.1	1.3	1.7	1.8
Pod shattering (1–9)	1.5	1.7	1.2	1.3	1.1	1.2
Final stand count (plants/m)	21.2	21.6	10.9	11.4	10.0	9.8
Grain moisture (%)	11.7	12.0	11.3	11.5	12.0	11.7
100 Seed weight (g)	18.2	17.8	13.0	13.0	18.0	18.0
Yield (t/ha)	3.5	3.6	2.6	2.6	3.5	3.5

^a,^^b,c^Varieties were in temperate, tropical, and subtropical germplasm, respectively

No significant differences were observed between SIP and control varieties at 0.05% significance level

**Table 4:** Plant characterization of insect protected soybean (SIP) compared to conventional control conducted in cultivated field trials in the USA and Brazil in accordance with China-specific regulatory submission considerations

Testing Regions	USA		Brazil	
	2018 (1 Site)^a^		2017/2018 (1 Site)^b^	
Plant Characteristics	SIP	Control	SIP	Control
Early stand count (plants/m)	13.0	12.6	12.2	13.1
Plant height V3 (cm)	14.2	15.1	11.0	11.7
Plant height V5 (cm)	21.2	19.6	20.9	21.1
Plant height R1 (cm)	37.4	36.9	52.2	51.7
Plant height R3 (cm)	85.4	88.2	127.9	123.6
Plant height R6 (cm)	96.2	93.0	127.0	125.2
Canopy coverage V3 (%)	32.5	30.0	16.3	16.3
Canopy coverage V5 (%)	30.0	30.0	53.8	53.8
Canopy coverage R1 (%)	56.3	55.0	88.8	88.8
Canopy coverage R3 (%)	100.0	100.0	100.0	100.0
Canopy coverage R6 (%)	100.0	100.0	100.0	100.0
Leaf number per plant V3	3.8	3.7	3.9	3.9
Leaf number per plant V5	4.8	4.7	5.8	6.0
Leaf number per plant R1	9.7	9.7	9.9	10.0
Leaf number per plant R3	13.5	15.0	15.1	14.3
Leaf number per plant R6	13.1	13.5	14.5	14.9
Final stand count (plants/m)	12.6	11.8	10.2	10.2
Seed shattering (%)	2.5	2.7	4.1	2.8
Seed number per plant	192.1	206.3	131.8	120.9
Shattered seed count (seeds/plot)	455.8	447.5	2232.5	1346.8
Volunteer plants (plants/plot)	0.0	0.0	1.5	2.0

^a,^^b^Varieties were in temperate and subtropical germplasm, respectively

No significant differences were observed between SIP and control varieties at 0.05% significance level

### Uncultivated field trials

Plant characterization was also conducted under uncultivated field trials following China-specific regulatory considerations with evaluations under different planting methods (row and surface), four different planting times, and assessment of both soybean and weed characteristics several times during the season (Tables 5 and 6).

**Table 5:** Characteristics of insect-protected soybean (SIP) compared to conventional control conducted in uncultivated field trials in the USA and Brazil in accordance with China-specific regulatory submission considerations

Comparisons	﻿Soybean characteristics									
	Stand count (plants/plot)^a^		Plant height (cm)^a^		Canopy coverage (%)^a^		Seed number per plant		Volunteer plants per plot	
	SIP	Control	SIP	Control	SIP	Control	SIP	Control	SIP	Control
*Planting method*										
﻿﻿Row	110.2 a	109.7 a	54.4 a	55.3 a	28.4 a	30.5 a	47.6 a	47.6 a	1.4 a	2.1 a
﻿Surface	12.3 b	14.8 b	38.0 b	39.0 b	7.1 b	﻿7.3 b	29.3 b	26.8 b	0.3 a	0.2 b
*Planting time*										
﻿First	96.2 a	98.7 a	58.8 a	59.3 a	35.8 a	36.7 a	63.4 a	62.0 a	0.1 b	0.2 a
﻿﻿Second	72.5 b	77.8 b	62.6 a	65.9 a	26.4 b	28.1 b	47.8 a	53.8 a	0.8 a	1.0 a
﻿ ﻿Third	50.9 c	49.3 c	37.3 b	37.6 b	6.3 c	7.8 c	20.8 b	15.5 b	1.1 ab	1.4 a
﻿﻿Fourth	25.4 d	23.1 d	30.1 b	30.0 b	2.3 d	2.8 d	19.2 c	14.5 c	1.4 ab	1.9 a
*Country*										
﻿﻿USA	39.7 b	39.6 b	37.7 b	37.1 b	13.1 b	14.9 b	23.8 b	24.5 b	0.0 b	0.1b
﻿﻿Brazil	82.7 a	84.9 a	54.7 a	57.0 a	22.3 a	22.8 a	52.3 a	49.6 a	1.7 a*	2.2a
*Average*	61.2	62.2	46.8	47.7	17.7	18.9	39.0	37.9	0.8	1.1

^a^Comparisons were done across four observation timings (30, 60, 90, and 120 days after planting)

For each column, the same letter indicates no significant differences among comparisons done for planting methods, planting times, or countries

^*^ Significant difference between SIP and control varieties at 0.05% significance level

**Table 6:** Characteristics of weeds grown in insect-protected soybean (SIP) and conventional control plots in uncultivated field trials in the USA and Brazil in accordance with China-specific regulatory submission considerations

Comparisons	﻿Weed characteristics^a^					
	Stand count (plants/plot)		Plant height (cm)		Canopy coverage (%)	
	SIP	Control	SIP	Control	SIP	Control
*Planting method*						
﻿Row	38.1 b	42.1 a	78.4 a	74.4 a	55.2 b	54.5 b
﻿﻿Surface	62.8 a	67.0 a	59.8 b	59.8 b	66.9 a	69.7 a
*Planting time*						
﻿ ﻿First	92.4 a	99.9 a	60.5 b	57.5 a	35.6 d	37.4 d
﻿﻿Second	37.7 ab	36.4 a	67.3 ab	67.3 a	51.8 c	52.3 c
﻿ ﻿Third	45.1 ab	52.0 a	73.4 ab	69.8 a	72.2 b	72.7 b
﻿﻿Fourth	26.5 b	30.0 a	74.5 a	72.7 a	84.9 a	86.0 a
*Country*						
﻿﻿USA	67.7 a	76.9 a	83.4 a	81.0 a	71.9 a	71.0 a
﻿﻿Brazil	33.1 a	32.2 b	55.5 b	53.9 b	50.3 b	53.2 b
*Average*	50.4	54.6	69.1	67.1	61.1	62.1

^a^Comparisons were done across five observation timings (pre-soybean planting, 30, 60, 90, and 120 days after planting)

For each column, the same letter indicates no significant differences among comparisons done for planting methods, planting times, or countries

No significant differences were observed between plots under SIP and control soybean at 0.05% significance level

**Fig. 1:**
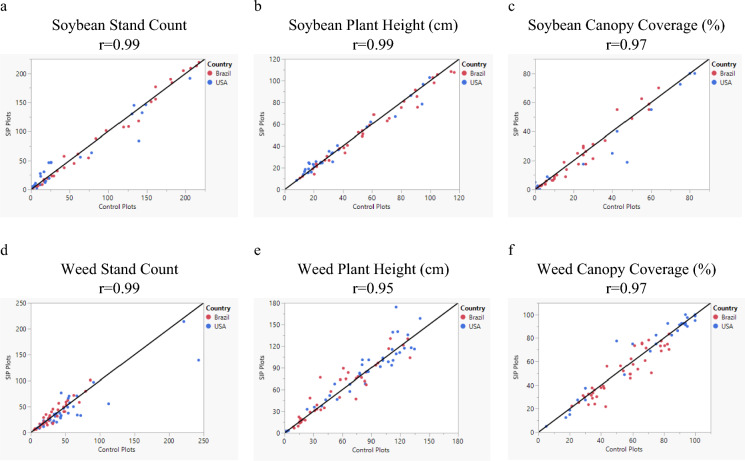
Correlations associated with plant characteristics between plots under insect-protected soybean (SIP) and conventional control soybean. Data collected in the USA (blue points) and Brazil (red points) in uncultivated field trials. Panels a, b, and c represent soybean characteristics, whereas d, e, and f represent weed characteristics. Pearson’s coefficient (r) was calculated for each characteristic. The black line in each graph represents the Y = X regression line

*Soybean Performance in Uncultivated Field Trials*: When averaged across observations, there were no significant differences between soybean with and without the SIP trait for any of the evaluated characteristics including stand count, plant height, canopy coverage, seed number, and volunteer plant number (Table 5). Furthermore, these characteristics showed comparable performance between soybean with and without the SIP trait regardless of planting methodology, planting time, or testing region. There was only one difference indicating that there are fewer volunteer plants observed for soybean with the SIP trait (1.7) compared to the conventional control (2.2) (Table 5). However, this variation in observed volunteer plants is not biologically meaningful as the difference was less than a single plant (a unit used for measuring this characteristic). Very high correlation coefficient values (r ≥ 0.97) were observed between soybean with and without the SIP trait for characteristics measured multiple times across the season (Fig. 1, panels a–c).

In these trials, significant differences were observed among different planting methods, planting times, or testing regions (Table 5). Generally, higher values were observed for soybean planted in rows than those scattered on the soil surface. Row planted soybean had substantially more plants that were taller with more canopy coverage which consequently produced more seeds compared to the plots where soybean seeds were scattered. Earlier planting resulted in more soybean plants, taller plants, more canopy coverage and in some cases more seeds. Furthermore, significant differences were observed between the USA and Brazil environments. Higher values for stand count, as well as taller plants, more canopy coverage, seeds, and volunteer plants were observed in trials conducted in Brazil.

**Fig. 2:**
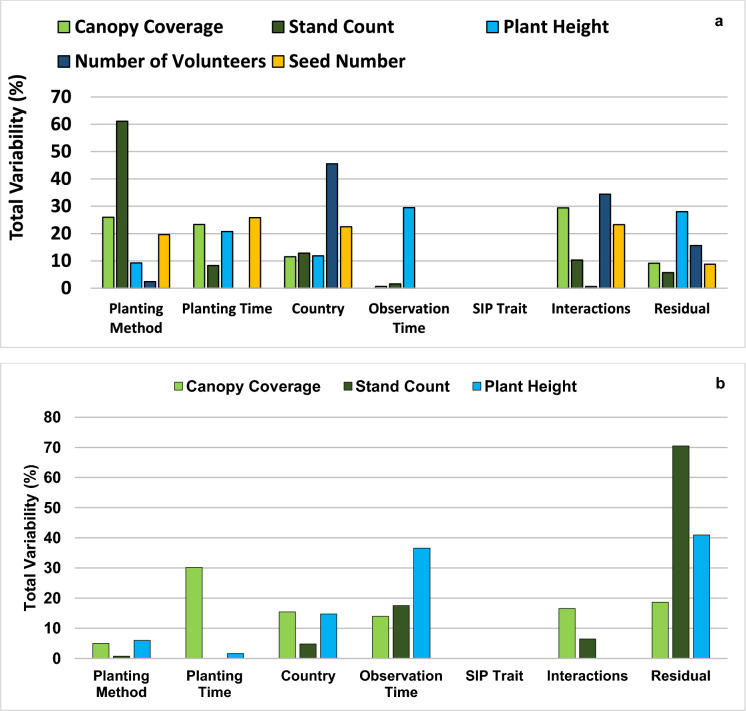
Percentages of total variability attributable to different factors (planting method, planting time, country, observation time, soybean insect-protected (SIP) event, interaction, and residual) for soybean (**a**) and weed (**b**) characteristics observed in uncultivated field trials

Variance component analysis was conducted to better understand how much different factors impacted the soybean characteristics evaluated. Stand count was impacted mostly by planting methodology, the number of volunteers was impacted predominantly by country (likely due to difference in climate conditions), whereas plant height was impacted most highly by observation timing. The analyses also indicated that the SIP trait did not contribute to variation for any of the evaluated characteristics (Fig. 2, panel a).

*Weed Performance in Uncultivated Field Trials*: When averaged across observations, there were no significant differences between weed performance in plots where soybean with and without the SIP trait was grown for any of the evaluated characteristics including stand count, plant height, and canopy coverage (Table 6). For these weed characteristics, very high correlation coefficient values (r ≥ 0.95) were observed between SIP and conventional control plots (Fig. 1, panels d–f).

In these trials, significant differences were observed among different planting methods, planting times, and testing regions (Table 6). Generally, higher weed stand count, shorter weed plants, and more weed canopy cover was observed in plots where soybean was scattered on the soil surface compared to where it was planted in rows. Earlier planting of soybean was associated with higher weed stand count, shorter weed plants, and less weed canopy cover compared to the plots where soybean was planted later. Higher values for weed stand count, taller weed plants, and more robust weed canopy coverage were observed in trials conducted in the USA compared to those grown in Brazil.

Variance component analysis indicated that the height of the weed plants was strongly impacted by observation timing, whereas the weed canopy coverage showed variation depending on planting time. The analyses also indicated that the presence of the SIP trait in soybean grown in these plots did not contribute to variation for any of the evaluated weed characteristics (Fig. 2, panel b).

## Discussion

Plant characterization under cultivated field trials was conducted using different germplasm (temperate, tropical, or subtropical), seasons (2012, 2013/2014, 2017/2018, and 2018), and testing regions (the USA and Brazil). These soybean trials followed either global regulatory requirements or China-specific considerations. Both approaches use comparative assessment of soybean with and without the SIP trait in the same genetic background. Regardless of the approach, both assessments showed no differences between soybean with and without the SIP trait for any of the evaluated characteristics. The values for a given characteristic might have differed depending on the germplasm, season, or testing region, however the conclusion regarding risk assessment of the SIP trait was the same. All trials showed comparable agronomic and phenotypic performance of soybean with and without the SIP trait.

Plant characterization under uncultivated field trials was conducted following China-specific regulatory considerations that included evaluations of soybean and weed characteristics under different planting methods, planting times, and observing characteristics several times during the season. These very complex and labor-intensive trials were based on a comparative assessment of the soybean variety with and without the SIP trait, as well as evaluating weed characteristics in SIP and control plots. Generally, comparable performance and very high correlation coefficients were observed between plots with and without the SIP trait for both soybean and weed characteristics regardless of planting methodology, planting time, or testing region. Furthermore, the variance component analyses indicated that the SIP trait did not contribute to variation for any of the characteristics evaluated to assess soybean or weed performance. The conclusion based on uncultivated field trials is similar to one based on results obtained from cultivated field trials. Namely, comparable performance of soybean with and without the SIP trait, as well as comparable performance of weeds observed in these plots. This occurred consistently in spite of the fact that soybean and weed performance in these trials was impacted by different planting methods, planting times, or testing regions.

As expected, soybean plants grew better when planted in rows and earlier in the season rather than those produced from scattered seeds or planted later. This is because soybeans planted in rows or earlier in the season had less challenges competing with weeds compared to those planted on surface or later. These results are not surprising as soybean is a highly domesticated crop that requires cultivation for better agronomic performance and competes poorly with weedy species.

Different values were observed for characteristics evaluated in the USA and Brazil. That is expected as soybean performs differently under temperate and subtropical environmental conditions. Factors other than climate (like agronomic practices, germplasm, weed pressure, and seasonal weather) also contributed to these differences. For example, lower soybean stand was due to higher weed pressure in the USA trials compare to that in Brazil. The subtropical variety grown in Brazil was taller compared to the temperate cultivar planted in the USA trials. Furthermore, these two trials were planted in two different seasons. As expected, these and other factors influenced differences observed in soybean performance between the two regions. However, regardless of differences associated with planting methods, planting times, or testing regions, soybean with and without the SIP trait had comparable performance, as did weed vegetation evaluated in SIP and control plots. This confirms that, even though the plant characterization values might be different for any given geographic location, the conclusions of comparative assessment are transportable across different regions. These results are in agreement with those reported by others (Bachman et al. 2021; Clawson et al. 2019; Matsushita et al. 2020; Nakai et al. 2015).

## Conclusion

In conclusion, results obtained from different regions, seasons, germplasm, and methodologies often had different plant characterization values, but each showed that the performance of soybean with the SIP trait is comparable to that of the conventional soybean. This indicates that soybean with the SIP trait does not differ from conventional soybean in its plant pest/weed potential. The ERA conclusion was consistent regardless of the testing approach. These results reinforce the concept of data transportability (across regions, seasons, germplasms, and methodologies) for comparative assessments and should be considered by global regulatory agencies in order to minimize redundancy and better harmonize approaches regarding ERA of biotechnology-derived products.

## Data Availability

All relevant data are shared in the paper.
